# Respiratory culture nudge improves antibiotic prescribing for *Moraxella catarrhalis* and *Haemophilus influenzae* lower respiratory tract infections

**DOI:** 10.1017/ash.2023.1

**Published:** 2023-02-02

**Authors:** Christen J. Arena, Rachel M. Kenney, Ronald E. Kendall, Robert J. Tibbetts, Michael P. Veve

**Affiliations:** 1 Department of Pharmacy, Henry Ford Health, Detroit, Michigan; 2 Department of Pharmacy, VA Ann Arbor Healthcare System, Ann Arbor, Michigan; 3 Department of Microbiology, Henry Ford Health, Detroit, Michigan; 4 Eugene Applebaum College of Pharmacy and Health Sciences, Wayne State University, Detroit, Michigan

## Abstract

We compared optimal antibiotic prescribing before and after implementing an interpretive β-lactamase microbiology comment for *Haemophilus influenzae* and *Moraxella catarrhalis* in lower respiratory-tract infections. The postintervention group was associated with 5-fold increased odds of optimal de-escalation (adjusted odds ratio, 5.03; 95% confidence interval, 2.57–9.87).

Lower respiratory-tract infections (LRTIs) are an important target for antimicrobial stewardship programs due to their frequency, diagnostic uncertainty, and association with antibiotic overuse; however, gaps in effective LRTI-targeted stewardship strategies remain. The development of pragmatic and impactful antimicrobial stewardship interventions are needed to improve optimal antibiotic utilization and patient outcomes.

Behavioral interventions such as nudging clinicians with purposeful, interpretative microbiology comments are associated with improved antibiotic prescribing.^3–6^ Notably, framing strategies with actionable comments is useful to direct antibiotic selection.^5^ In 2 studies utilizing purposeful microbiology report comments, significant improvement was achieved in overall appropriateness of antibiotic use.^3,5,6^

The Clinical Laboratory Standards Institute recommends a direct β-lactamase test as a means of rapid identification for ampicillin or amoxicillin resistance.^7^ Laboratory reports for *Hemophilus influenzae* and *Moraxella catarrhalis* may be perceived as ambiguous to prescribers unfamiliar with the meaning of a positive or negative β-lactamase result. To minimize ambiguity and to assess optimal definitive antibiotic prescribing, we implemented a nudge with an automated, interpretive respiratory culture comment for β-lactamase negative or positive *H. influenzae* or *M. catarrhalis* LRTIs. We hypothesized that implementation of a targeted microbiology nudge would be associated with optimal de-escalation in this setting.

## Methods

### Study design

This before-and-after quasiexperimental study was conducted at Henry Ford Health over 2 periods: a preintervention period before the microbiology comment (from August 20, 2017, to March 19, 2019) and a corresponding postintervention period after the microbiology comment was implemented (from March 21, 2019, to August 20, 2021). Hospitalized patients were included if they were aged ≥18 years and had been prescribed antibiotics for an LRTI with dominant growth of *H. influenzae* or *M. catarrhalis*. Respiratory specimens included in the study were sputum, tracheal aspirate, and bronchoalveolar lavage (BAL). Patients were excluded for non-LRTI or polymicrobial infections, receipt of prophylactic antibiotics for opportunistic infections, and documented IgE-mediated β-lactam allergy. Only the index episode of *H. influenzae* or *M. catarrhalis* LRTI was included. The study received institutional review board approval with waiver of consent.

### Intervention

On March 20, 2019, an automated, interpretive comment on respiratory cultures for β-lactamase negative or positive *H. influenzae* or *M. catarrhalis* was implemented. The intervention consisted of appending a new comment for β-lactamase negative *H. influenzae* or *M. catarrhalis* stating, “This organism is predictably susceptible to ampicillin or amoxicillin.” For β-lactamase positive *H. influenzae* or *M. catarrhalis*, the new comment stated, “This organism is predictably susceptible to ampicillin-sulbactam or amoxicillin-clavulanate.” These results are available within patient culture results in the electronic medical record (EMR). Prior to the intervention, microbiology reports stated the β-lactamase result as either “positive” or “negative” with no interpretation.

### Study data and outcome definitions

Patient and infection-related characteristics were manually collected from the EMR using a standardized case report form. Microbiology variables included were infection type, duration of antibiotic therapy, and *Clostridioides difficile* infection after initial antibiotic therapy until discharge. Identification of *M. catarrhalis* or *H. influenzae* from respiratory isolates was determined by the Henry Ford Health centralized clinical microbiology laboratory using BD BBL Cefinase paper disk β-lactamase testing (BD Microbiology Systems, Cockeysville, MD).

The primary outcome was optimal de-escalation, defined as definitive therapy with ampicillin or amoxicillin for β-lactamase–negative isolates and amoxicillin-clavulanate or ampicillin-sulbactam for β-lactamase–positive isolates. Secondary outcomes included de-escalation based on specimen type, clinical success, duration of treatment in days, time to de-escalation in days, and continuation of broad-spectrum antibiotics after final microbiology results. Clinical success was defined as improvement in signs and symptoms of infection without need for further antibiotics after completion of the index course. A nonequivalent dependent variable of deep-vein thrombosis (DVT) prophylaxis was used to evaluate overall quality of medication management over the study period to assess maturation.

### Statistical analyses

A sample size of 201 patients was calculated using the following assumptions: (1) 2-sided α of 0.05; (2) β of 0.8; and (3) and anticipated effect size of 30% change in optimal de-escalation after comment implementation.^3^

In bivariate analysis, continuous data were analyzed using the Mann-Whitney U test; categorical data were compared using the χ^2^ or Fisher exact test. Stratified analyses by organism and infection type were performed to assess the impact of effect modifiers. To determine patients with the greatest likelihood of receiving optimal antibiotic de-escalation, variables associated with the outcome (*P* < .20) from bivariate analysis were entered into a multivariable model using a backward, stepwise approach. All statistical tests were performed using SPSS software (SPSS Statistics for Windows version 26.0, Armonk, NY; IBM Corp).

## Results

In total, 201 patients were included: 100 in the preintervention group, 101 in the postintervention group. Baseline characteristics are presented in Table 1. The overall organism distribution was 159 (79%) *H. influenzae* and 49 (24%) *M. catarrhalis*; 7 cultures grew both organisms with the same β-lactamase result.

**Table 1.** Baseline and Infection Characteristics of Patients With *Haemophilus influenzae* or *Moraxella catarrhalis* Lower Respiratory Tract Infections Before and After Implementation of an Interpretive β-lactamase Comment

Baseline Characteristics	Before the Intervention (*n* = 100), No. (%)	After the Intervention (*n* = 101), No. (%)	*P* Value
Age, median years (IQR)	63 (42–84)	63 (46–80)	.40
Sex, male	61 (61)	65 (64)	.62
PWID	7 (7)	5 (5)	.54
β-lactam allergy (non-IgE mediated)	4 (4)	7 (7)	.36
**Infection characteristics**			
CAP	63 (63)	74 (73)	.12
HAP	20 (20)	15 (15)	.34
VAP	17 (17)	12 (12)	.30
*H. influenzae*	81 (81)	78 (77)	.51
*M. catarrhalis*	24 (24)	25 (25)	.90
Positive β-lactamase	46 (46)	52 (52)	.44
ID consult	16 (16)	18 (18)	.73
Antibiotic duration >7 days	36 (36)	33 (33)	.62
**Severity of illness**			
CCI, median (IQR)	4 (3–8)	4 (2–6)	.12
WBC >12 or <4 per µL	55 (55)	50 (50)	.44
Heart rate >90 bpm	49 (49)	46 (46)	.62
RR >20 bpm or pCO_2_ <32	46 (46)	36 (36)	.22
Temperature >38.3°C	17 (17)	20 (20)	.61
Pressors at time of culture collection	27 (27)	27 (27)	1.0
Ventilated at time of culture collection	57 (57)	55 (55)	.72

Note. PWID, persons who inject drugs; CAP, community-acquired pneumonia; HAP, hospital-acquired pneumonia; VAP, ventilator-associated pneumonia; ID, infectious diseases; CCI, Charlson comorbidity index; WBC, white blood cell; RR, respiratory rate.

The primary outcome of optimal de-escalation occurred in 19 patients (19%) in the preintervention group and 51 patients (51%) in the postintervention group (*P <* .001). The proportions of patients who received DVT prophylaxis, the nonequivalent dependent variable, in the preintervention and postintervention groups were 93 (93%) and 82 (81%) (*P* = .013). After adjusting for persons who inject drugs (PWID), hospital-acquired pneumonia (HAP), and patients with sputum cultures, the β-lactamase comment was associated with a 5-fold increased odds of optimal de-escalation (adjOR, 5.03; 95% CI, 2.57–9.87) (Table 2). In total, optimal de-escalation occurred in 50 patients (50%) with sputum, 39 (39%) with tracheal aspirate, and 11 (11%) with BAL cultures (*P* = .07). The median time to de-escalation, in days, was 1 (IQR, 0–2) day in the preintervention group and 0 (IQR, 0–1) days in the postintervention group (*P* < .001). The total median duration of antibiotic therapy was 7 days (IQR, 6–8) in the preintervention group and 7 days (IQR, 6–10) in the postintervention group (*P* = .28).

**Table 2.** Variables Associated With Optimal De-escalation

Variable	Optimal De-escalation, No. (%)	Unadjusted OR (95% CI)	*P* Value	Adjusted OR (95% CI)
Postintervention β-lactamase nudge	51 (73)	4.35 (2.31–8.20)	<.001	5.03 (2.57–9.87)
PWID	1 (1)	0.16 (0.02–1.25)	.06	0.15 (0.02–1.22)
HAP	16 (23)	1.75 (0.83–3.66)	.137	2.17 (0.96–4.92)
Sputum sample	35 (50)	0.58 (0.32–1.04)	.067	0.48 (0.25–0.92)
Receipt of vasopressor therapy	20 (29)	1.16 (0.61–2.23)	.65	Not tested
ID consultation	13 (19)	1.20 (0.56–2.56)	.65	Not tested

Note. PWID, persons who inject drugs; HAP, hospital-acquired pneumonia; ID, infectious diseases; OR, odds ratio; CI, confidence interval.

Hosmer Lemeshow goodness of fit, 0.727.

In total, 79 patients (79%) met the definition of clinical success in the preintervention group, compared to 81 patients (81%) in the postintervention group (*P* = .54). All-cause, in-hospital mortality occurred in 15 patients (15%) in the preintervention group and 9 patients (9%) in the postintervention group (*P* = .18). *C. difficile* infection occurred in 4 patients (4%) in the preintervention group and 2 patients (2%) in the postintervention group (*P* = .45).

## Discussion

Optimal de-escalation significantly improved after implementation of an interpretive β-lactamase comment, with no negative impact on patient outcomes. Our findings suggest that prescribers may be unfamiliar with the meaning of β-lactamase results for *M. catarrhalis* and *H. influenzae* and that they are more likely to act in response to a purposeful comment. This unfamiliarity could be related to the misconception that all β-lactamase–producing organisms reflect resistance requiring broad-spectrum antibiotic therapy (ie, carbapenem). These data add to the body of literature on nudging providers into action using a framing microbiology comment.^3–6^

This study had several limitations. This study may have been subject to maturation bias and/or regression to the mean. To evaluate for maturation, we used a nonequivalent dependent variable of DVT prophylaxis to assess quality of medication management, which was not improved in the intervention group. LRTI specimen quality can also be considered a limitation because this could influence prescriber comfort with de-escalation. The capacity of an institution to adopt microbiology-culture comment nudges may be a limiting factor in generalizability to other centers without advanced microbiology services.

Microbiology nudges are pragmatic, sustainable stewardship interventions that can be reproduced in most settings without substantial resources.^3,10^ An interpretative β-lactamase comment for *M. catarrhalis* and *H. influenzae* was associated with improved definitive therapy prescribing. This study supports nudging within the EMR to positively influence antibiotic prescribing.
